# Strategies for Molecularly Enhanced Chemotherapy to Achieve Synthetic Lethality in Endometrial Tumors with Mutant p53

**DOI:** 10.1155/2013/828165

**Published:** 2013-12-07

**Authors:** Xiangbing Meng, Don S. Dizon, Shujie Yang, Xinjun Wang, Danlin Zhu, Kristina W. Thiel, Kimberly K. Leslie

**Affiliations:** ^1^Department of Obstetrics and Gynecology, University of Iowa Hospitals and Clinics, Iowa City, IA 52242, USA; ^2^Holden Comprehensive Cancer Center, University of Iowa, Iowa City, IA 52242, USA; ^3^Gillette Center for Gynecological Oncology, Massachusetts General Hospital Cancer Center, Boston, MA 02114, USA

## Abstract

Serous uterine endometrial carcinomas are aggressive type II cancers with poor outcomes for which new treatment strategies are urgently needed, in particular, strategies that augment sensitivity to established chemotherapy regimens. The tumor suppressor gene *TP53* is dysregulated in more than 90% of serous tumors, altering master regulators of the G2/M cell cycle checkpoint in unique and predictable ways and desensitizing cells to chemotherapy. We hypothesized that synthetic lethality can be achieved in endometrial cancer cells with mutant p53 by combining paclitaxel with agents to overcome G2/M arrest and induce mitotic catastrophe. The combination of BIBF1120, an investigational VEGFR, PDGFR, and FGFR multityrosine kinase inhibitor with established anti-angiogenic activity, with paclitaxel abrogated the G2/M checkpoint in p53-null endometrial cancer cells via modulation of G2/M checkpoint regulators followed by induction of mitotic cell death. In endometrial cancer cells harboring an oncogenic gain-of-function p53 mutation, synthetic lethality was created by combining paclitaxel with BIBF1120 and a histone deacetylase inhibitor, which serves to destabilize mutant p53. These cells were also sensitive to an inhibitor of the G2/M kinase Wee1 in combination with paclitaxel. These findings reveal that, in addition to antiangiogenic activity, the angiokinase inhibitor BIBF1120 can be used to restore sensitivity to paclitaxel and induce mitotic cell death in endometrial cancer cells with non-functional p53. These preclinical data serve as a critical platform for the creative design of future clinical trials utilizing molecularly enhanced chemotherapy to achieve synthetic lethality based on the mutational landscape.

## 1. Introduction

While outcomes have substantially improved for many types of cancer, endometrial cancer incidence and deaths are on the rise, with the five-year survival rate being worse today than three decades ago [1]. Inadequate sensitivity to chemotherapy is a primary cause of therapeutic failure. In addition, the promise of targeted therapy with molecular inhibitors in combination with chemotherapy, now in phase III trials for the treatment of other forms of cancer, is in its infancy in this disease. Though recent studies of single agents such as the vascular endothelial growth factor (VEGF) inhibitor bevacizumab have yielded the first molecular therapies deemed “clinically active” in endometrial cancer [2], these studies were performed in patients with advanced or recurrent disease that had progressed after chemotherapy, and response rates were modest. Thus, it is clear that molecular therapies cannot be used alone or restricted to patients with advanced/recurrent disease who have failed chemotherapy. In order to improve patient outcomes, there is a critical need to identify strategies to restore chemosensitivity and increase efficacy of these agents. Towards that goal, bevacizumab was the first antiangiogenic agent to significantly prolong progression-free survival (PFS) when combined with carboplatin and paclitaxel as compared to chemotherapy alone for the treatment of epithelial ovarian cancer [3, 4]. Other investigational antiangiogenic agents, such as the triple angiokinase inhibitor BIBF1120 (also nintedanib; targets receptors for VEGF, fibroblast growth factor (FGF), and platelet-derived growth factor (PDGF)), have been studied in phase III maintenance therapy trials. These trials evaluated PFS as the primary endpoint (i.e., the AGO-OVAR12/LUME-Ovar1 trial) [4, 5]. Use of these angiokinase inhibitors may be superior to traditional tyrosine kinase inhibitors because they will target not only the signaling pathways that tumors rely on for survival, but also the tumor vasculature. However, we still must understand which tumors will respond and which will not.

One strategy is to identify Achilles' heels provided by distinct mutations within each tumor. Endometrial adenocarcinomas are broadly divided into two types based on histologic features: type I endometrioid adenocarcinomas and type II serous adenocarcinomas. Endometrioid endometrial cancer, a typically lower histological grade disease, is associated with gene mutations in *PTEN* (50%–83%), PI3Kinase (*PIK3CA, R1* and* R2* 40%–80%), *KRAS* (20%), and *FGFR2* (12%) and microsatellite instability (20%), while *TP53* mutations appear to be the key driver in serous lesions (~90% of nonendometrioid lesions) [6–9]. It is critically important to note that varying types of p53 mutant proteins exist, with different implications for chemosensitivity. Some mutations are relatively inconsequential from the perspective of p53 function, and proteins of this type retain wild-type activity. Other mutations are loss-of-function (LOF) in which single amino acid changes completely inactivate or destabilize the protein. Finally, an interesting category is the gain-of-function (GOF) p53 mutations that convert p53 from a tumor suppressor to an oncogene. Substantial clinical and preclinical data from a wide range of cancers indicate that GOF p53 mutations predict a poor response to treatment [10–12], though limited data are available for tumors of the endometrium.

In response to DNA damaging agents such as carboplatin and doxorubicin, cells activate a checkpoint signaling pathway downstream of ATM/ATR using effectors Chk1/Chk2 and a more recently identified branch through p38/MK2 in order to arrest cell cycle progression and repair DNA [13–15]. Chk1, Chk2, p53, and MK2 maintain the checkpoint by inhibiting the CDC25 phosphatases (activators of Cyclin B/Cdc2 in mitosis) [16–18]. The ability of cells to activate cell cycle checkpoints prevents progression into vulnerable phases of the cell cycle, M for paclitaxel and S for carboplatin and doxorubicin, leading to chemoresistance. The newly identified branch of cell cycle control via p38/MK2 is particularly relevant in endometrial cancer and is activated by the most common mutations driving this disease.

In cells with LOF p53, p38/MK2 and downstream components ultimately controlling the critical phosphatase CDC25C and Cyclin B/Cdc2 are activated as an alternative means to maintain the checkpoint [15]. We now understand that this pathway can be coopted by oncogenic alterations including p53 GOF (oncogenic) mutants and activated Ras mutants [19, 20]. Constitutive activation of p38 and downstream MK2 lead to an inhibitory phosphorylation event on the phosphatase CDC25C, inhibition of Cdc2, G2/M checkpoint maintenance, and chemoresistance. Polo-like kinase 1 (PLK1), which is upregulated in many cancers, plays a pivotal role in all phases of mitosis [21]. PLK1 is downregulated at the transcriptional level by p53 as part of the G2/M checkpoint [22–26]. Thus, p53 null cells are unable to downregulate PLK1 in response to chemotherapy, leading to chemoresistance [25, 26]. Indeed, PLK1 colocalizes with p38 and MK2 at the spindle during mitosis and is phosphorylated by MK2, linking their activities and suggesting pathway crosstalk [27].

We recently made the important discovery that endometrial cancer cells with inactivated p53 rely on the p38 pathway to maintain the G2/M checkpoint [28]. As such, these p53-null tumors are exquisitely sensitive to the combination of epidermal growth factor receptor (EGFR) inhibitor gefitinib with paclitaxel, which abrogates the G2/M checkpoint. Specifically, treatment of p53-null endometrial cancer cells with gefitinib lowered the IC50 of paclitaxel by 10-fold, with a combination index of 0.25 indicative of profound synergy. Since endometrial cancer cells express multiple angiogenic tyrosine kinase receptors, the objective in this study was to determine whether anti-angiogenic agents can be used to achieve synthetic lethality in combination with paclitaxel in p53 mutant endometrial cancer cells.

## 2. Materials and Methods

### 2.1. Reagents

All antibodies were purchased from Cell Signaling. Gefitinib (ZD1839, Iressa, AstraZeneca) and paclitaxel were suspended in dimethyl sulfoxide (DMSO). BIBF1120 (nintedanib), LBH589, and MK-1775 (Selleck Chemicals) were suspended in DMSO.

### 2.2. Cell Lines and Culture Conditions

Hec50co endometrial cancer cells, a subline of Hec50 cells, were kindly provided by Dr. Erlio Gurpide (New York University). Paclitaxel-resistant Hec50 cells, Hec50A and Hec50E, were obtained from parental Hec50co cells grown as xenograft tumors in mice as previously described [28]. KLE cells were purchased from ATCC. Cells were cultured in DMEM (Sigma-Aldrich) with 10% fetal bovine serum (Gemini Bio-Products) and 2 mM L-glutamine (Invitrogen).

### 2.3. Expression of p53 in Hec50 Cells

To generate p53 R175H GOF mutant, a vector containing wild-type p53 cDNA (Clontech) was subjected to site-directed mutagenesis (Stratagene) per manufacturer's instructions. Hec50 cells were transfected with constructs containing either WT or R175H p53 using Lipofectamine 2000 as per manufacturer's instructions (Invitrogen). Individual cell clones were selected for resistance to G418, expanded, and screened for p53 expression by Western blotting.

### 2.4. Western Blot Analysis

As previously described [28], cells were plated in 100 mm dishes and were allowed to grow for 24 h prior to treatment. After treatment for 24 h, cells were harvested, lysed with extraction buffer (1% Triton X-100, 10 mM Tris-HCl pH 7.4, 5 mM EDTA, 50 mM NaCl, 50 mM NaF, 20 *μ*g/mL aprotinin, 1 mM PMSF, and 2 mM Na_3_VO_4_), and subjected to three freeze/thaw cycles as previously described [28]. Equal amounts of protein (determined by the method of Bradford, BioRad) were subjected to SDS-PAGE followed by transfer to nitrocellulose membranes (BioScience). Membranes were probed with primary antibodies against *β*-actin, p53, phospho-cdc2 Tyr15, CDC25C, phospho-Wee1 Ser642, phospho-Myt1 Ser83, phospho-stathmin Ser38, total stathmin, and phospho-histone H3 Ser10 followed by incubation with corresponding horseradish peroxidase-conjugated secondary antibody. The signal was visualized by chemiluminescence using ECL Western blotting detection reagents (Pierce).

### 2.5. Cell Cycle Analysis by Flow Cytometry

Cells were plated in 100 mm dishes with an equal number of cells in each dish and treated for 24 h. Cells were fixed in 70% ethanol. After washing with PBS, cells were incubated in Krishan's solution (3.8 mM sodium citrate, 0.014 mM propidium iodide, 1% NP-40, and 2.0 mg/mL RNase A) for 30 minutes at 37°C and analyzed by FacScan Flow Cytometer (Becton, Dickinson and Company) as previously described [28]. The data were subjected to further analysis by CellQuest software version 3.3, which generated DNA histograms indicating the fractions of the cell population in the sub-G1, G0-G1, S, or G2/M phase of the cell cycle. Experiments were performed in triplicate.

### 2.6. Quantitation of Percentage of Mitotic Cells

Cells were plated in 100 mm dishes with an equal number of cells and treated for 24 h. For metaphase spreads, cells were fixed with methanol : acetic acid (3 : 1). For visualization, cells were pipetted onto glass slides and stained with 4′,6-diamidino-2-phenylindole (DAPI). Mitotic spreads were viewed and imaged using fluorescence microscopy. The percentage of cells in mitosis was manually counted. A total of 300 cells for each treatment group were analyzed from three independent experiments.

### 2.7. Cell Viability Assays

Beginning 24 h after plating equal numbers of cells, cells were treated for 72 h followed by assessment of cell viability using the Wst-1 assay per manufacturer's instructions (Clontech). Data were quantitated relative to values obtained for control cells, which were set at 100% viability.

## 3. Results

### 3.1. BIBF1120 Increases Sensitivity to Paclitaxel in p53-Null Parental and Paclitaxel-Resistant Hec50 Cells by Producing a High Percentage of Mitotic Cells

**Figure 1:**
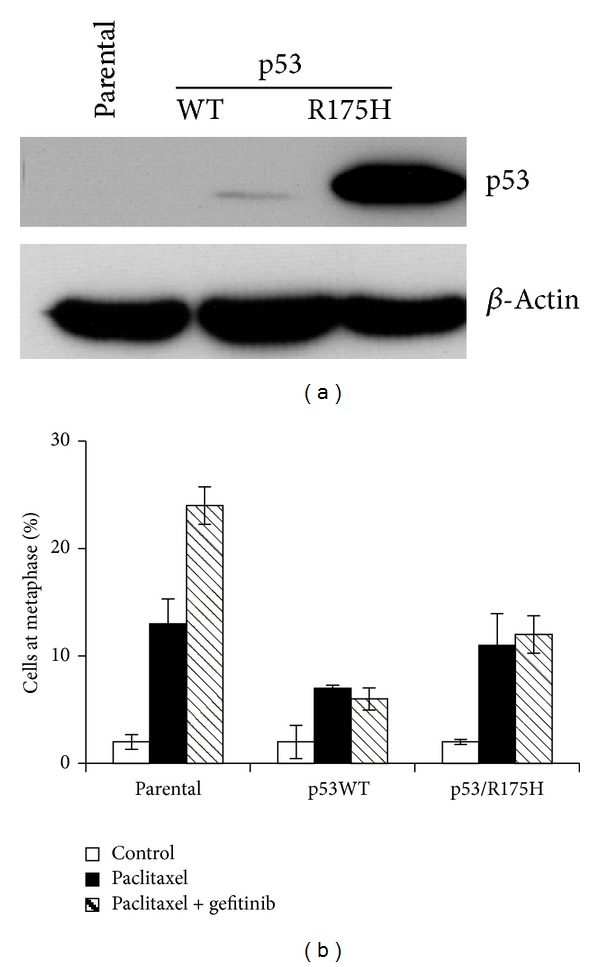
Synthetic lethality to paclitaxel + RTK inhibitor in endometrial cancer cells requires loss of functional p53. Hec50 endometrial cancer cells which are p53-null were transfected with either WT p53 or GOF p53 mutant R175H, one of the most commonly observed p53 GOF mutations in cancer. (a) Expression of p53 in Hec50 cells by Western blotting. (b) Percentage of mitotic cells after treatment with paclitaxel (10 nM) and/or EGFR inhibitor gefitinib (10 *μ*M) for 24 h.

Building on our recent study in which we achieved synthetic lethality by combining paclitaxel with the EGFR inhibitor gefitinib in p53-null endometrial tumors [25], we first sought to verify that the loss of p53 function is required for this effect. The p53-null poorly differentiated aggressive Hec50 endometrial cancer cells were transfected with either wild type (WT) p53 or R175H p53 GOF mutant. As anticipated [29], levels of WT p53 were very low in the absence of DNA damage, whereas the R175H p53 GOF mutant was very stable (Figure 1(a)). Expression of WT or GOF p53 prevented induction of mitotic arrest with paclitaxel and gefitinib as evidenced by significantly fewer cells in mitosis as compared to parental p53-null cells treated with this regimen (Figure 1(b)). These data validate the requirement for nonfunctional p53 to achieve synthetic lethality.

**Figure 2:**
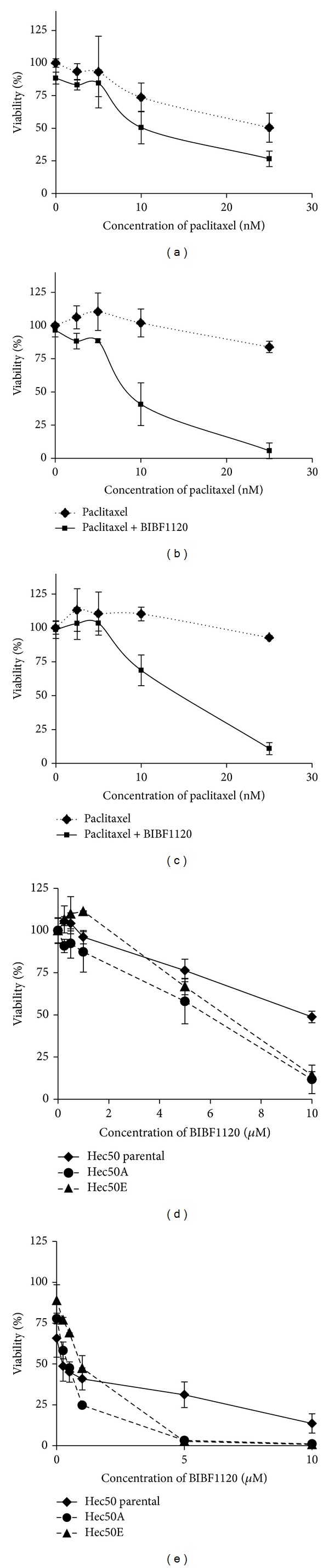
Angiokinase inhibitor BIBF1120 increases sensitivity to paclitaxel in parental and paclitaxel-resistant Hec50 cells. (a–c) Parental Hec50 (a) or paclitaxel-resistant Hec50A (b) or Hec50E (c) endometrial cancer cells, which are p53-null, were treated with increasing concentrations of paclitaxel in the absence or presence of 1 *μ*M BIBF1120 for 72 h, followed by assessment of cell viability using the Wst-1 assay. (d, e) Parental Hec50 or paclitaxel-resistant Hec50A or Hec50E endometrial cancer cells were treated with increasing concentrations of BIBF1120 in the absence (d) or presence (e) of 10 nM paclitaxel for 72 h, followed by assessment of cell viability using the Wst-1 assay.

The use of gefitinib and paclitaxel represents the first-generation approach for synthetic lethality in p53-null endometrial tumors. Given that any strategy that inhibits activation of p38 should theoretically induce synthetic lethality when combined with paclitaxel in p53-null cells, we next explored the use of triple angiokinase inhibitor BIBF1120, which not only inhibits VEGFR, PDGFR, and FGFR2, but also has antiangiogenic activity in the vascular endothelium. First, we established endometrial cancer cell lines that are highly resistant to paclitaxel. Parental Hec50 cells were grown as xenograft tumors in nude mice and treated with the PLK1 inhibitor BI2536 [28]. Tumors which did not respond to BI2536 were excised and cultured; these cell lines are referred to as Hec50A and Hec50E [28]. As compared to the parental Hec50 cells, Hec50A and Hec50E were extremely resistant to paclitaxel (Figure 2). However, synthetic lethality could be achieved in both the parental and the paclitaxel-resistant cells by the addition of 1 *μ*M angiokinase inhibitor, BIBF1120, which targets VEGFR, PDGFR, and FGFR2 (Figure 2).

**Figure 3:**
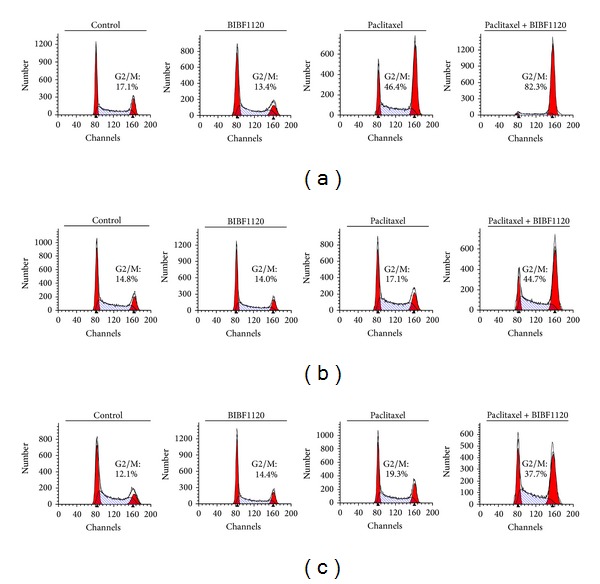
Combination of BIBF1120 and paclitaxel produces a profound increase in the percentage of cells in G2/M. Cell cycle profiles of parental (a) and paclitaxel-resistant Hec50A (b) and Hec50E (c) cells after treatment with 1 *μ*M BIBF1120, 14 nM paclitaxel, or the combination for 24 h. The percentage of cells in G2/M is indicated in each plot.

**Figure 4:**
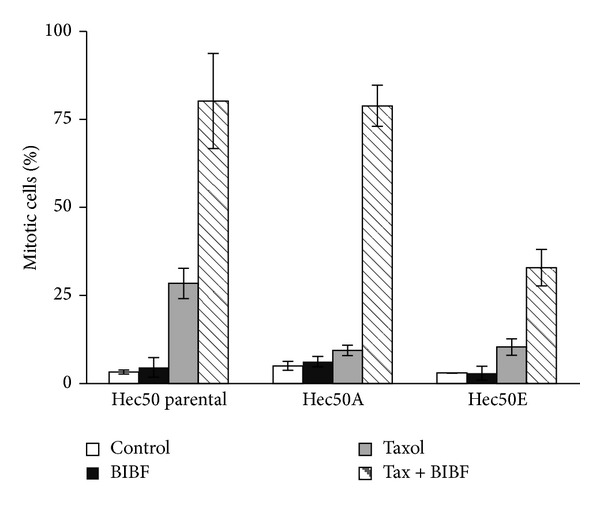
BIBF1120 and paclitaxel increase the percentage of mitotic parental and paclitaxel-resistant Hec50co cells. Parental Hec50 (a) or paclitaxel-resistant Hec50A (b) or Hec50E (c) endometrial cancer cells were treated with 14 nM paclitaxel for 24 h with or without 1 *μ*M BIBF1120, followed by assessment of the percentage of mitotic cells.

We next performed flow cytometry analysis to examine the effect of paclitaxel, BIBF1120, and the combination on cell cycle distribution. The percentage of cells in G2/M at baseline was similar among parental and paclitaxel-resistant Hec50 cells (Figure 3). Similarly, treatment with BIBF1120 alone had no effect on the percentage of cells in G2/M (Figure 3). We previously established that the IC50 of paclitaxel is 14 nM in parental Hec50 [28]. When the parental cells were treated with paclitaxel at 14 nM, there was a substantial increase in cells in G2/M (17% for control and 46% for paclitaxel, Figure 3(a)). Consistent with resistance to paclitaxel, the percentage of Hec50A and Hec50E cells in G2/M was unchanged with paclitaxel treatment as compared to control (Figures 3(b) and 3(c)). By contrast, the combination of paclitaxel and BIBF1120 produced a profound increase in the accumulation of cells in G2/M. We next examined the percentage of cells in mitosis. In the parental Hec50 cells and the paclitaxel-resistant cells, the combination of BIBF1120 and paclitaxel resulted in arrest in M phase (Figure 4), though the effect was dampened in Hec50E cells. This may be due to different mechanisms underlying resistance to paclitaxel in these clones, which may also impact sensitivity to the combination treatment at a particular dose. Consistent with this notion, viability studies in Figure 2 demonstrate that Hec50E cells require a slightly higher concentration of paclitaxel to reach an IC50 in combination with BIBF1120. Taken together, these data indicate that the combination of BIBF1120 and paclitaxel results in mitotic arrest and synergistic cell death.

### 3.2. Effect of Paclitaxel and BIBF1120 Combination Treatment on G2/M Cell Cycle Regulators

**Figure 5:**
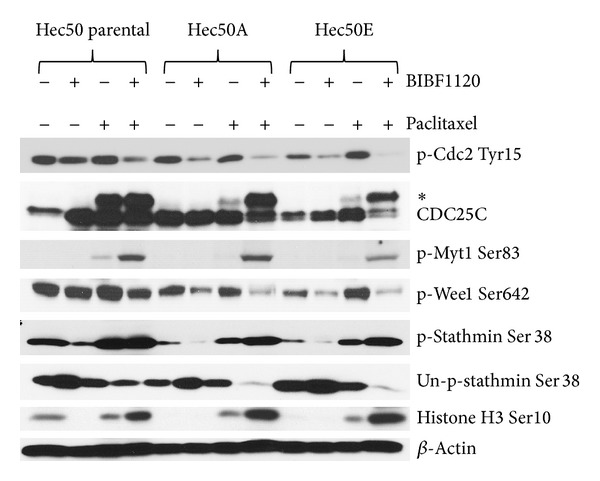
BIBF1120+paclitaxel modifies G2/M checkpoint controllers to induce transition into M phase. The effect of 1 *μ*M BIBF1120, 14 nM paclitaxel, and combination treatment (24 h) on the posttranslational modification of cell cycle regulators was examined by Western blotting. *denotes slower-migrating CDC25C, indicative of activating phosphorylation.

We next examined expression and activation of critical regulators of the G2/M checkpoint. The combination of BIBF1120 and paclitaxel resulted in activation of Cdc2 as evidenced by decreased phosphorylation at Tyr15 (Figure 5). The active form of CDC25C, a phosphatase that activates Cdc2 by dephosphorylating Tyr15, was significantly increased in cells treated with the combination of paclitaxel and BIBF1120 as demonstrated by a slower-migrating band compared to control or either drug alone (Figure 5). We also examined activation of other kinases, Wee1 and Myt1, that phosphorylate Cdc2 at Tyr15 to maintain Cdc2 in an inactive state. Wee1 phosphorylation at Ser642 is indicative of activation, whereas Myt1 phosphorylation at Ser83 reflects an inactive kinase. The combination treatment resulted in a decrease in phosphorylation of Wee1 at Ser642 and an increase in phosphorylation of Myt1 at Ser83, suggesting that both kinases are inactivated by BIBF1120 and paclitaxel. Consistent with the activation of Cdc2, treatment with paclitaxel and BIBF1120 promoted phosphorylation of stathmin-1 (STMN1), a microtubule destabilizer that is inactivated when phosphorylated. Finally, paclitaxel and BIBF1120 combination treatment resulted in a significant increase in phosphorylation of the histone H3 at Ser10, an established marker for mitosis. These data provide compelling evidence that the mechanism by which BIBF1120 induces synthetic lethality to paclitaxel is through abrogation of the G2/M checkpoint.

### 3.3. Strategies to Induce Synthetic Lethality in Cells with p53 GOF Mutation

**Figure 6:**
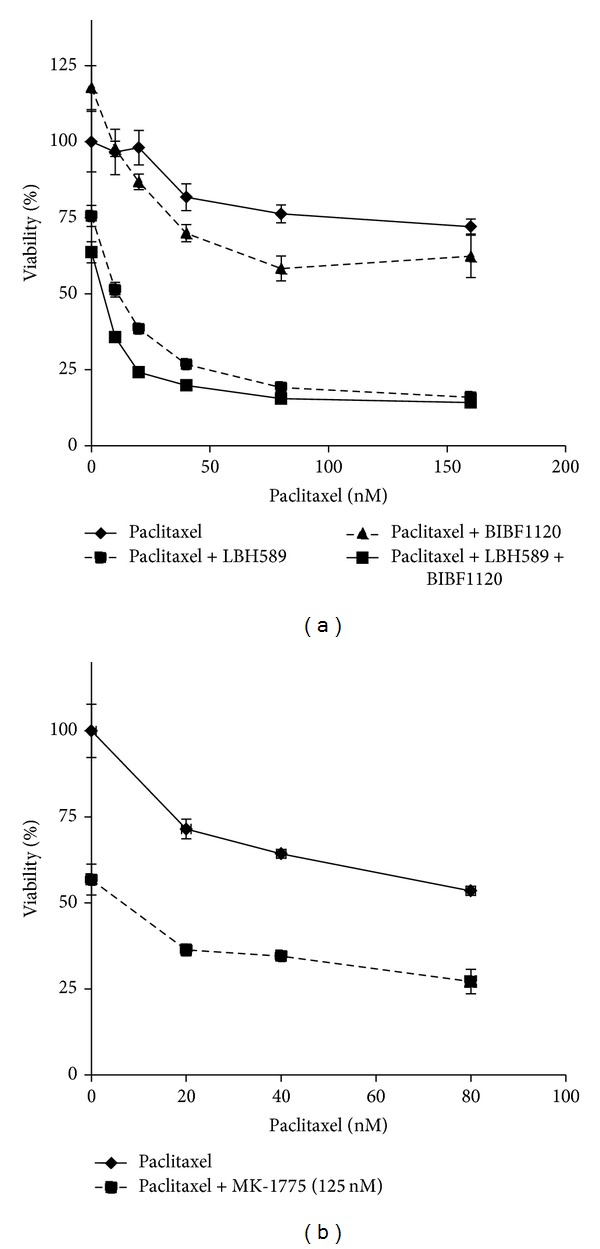
Strategies to achieve synthetic lethality in cells with p53 GOF mutation. (a) KLE endometrial cancer cells, which contain R175H p53 GOF mutation, were treated with increasing concentrations of paclitaxel in the presence of 10 nM LBH589 −/+ BIBF1120 (1 *μ*M) for 72 h, followed by assessment of cell viability by Wst-1 assay. (b) KLE cells were treated with increasing concentrations of paclitaxel −/+ Wee1 inhibitor MK-1775 (125 nM) for 72 h, followed by assessment of cell viability.

Our preliminary data indicate that 15%–20% of serous tumors harbor p53 GOF mutations (Leslie, unpublished observations), which can lead to hyperactivation of the p38 pathway and resistance to gefitinib and paclitaxel combination therapy [28]. We first examined whether KLE endometrial cancer cells that contain a mutant p53 are also resistant to the combination of BIBF1120 and paclitaxel. As shown in Figure 6(a), addition of BIBF1120 to paclitaxel had no appreciable impact on cell viability. It has been reported that p53 mutants associate with the heat shock protein 90 (Hsp90) machinery, which serves to stabilize the mutated p53 protein [29, 30]. The interaction between the heat shock protein and its client proteins can be disrupted by acetylation of Hsp90 [31]. Therefore, we hypothesize that treatment with the HDAC inhibitor (HDACi) LBH589 has the potential to cause dissociation of the GOF p53-Hsp90 complex, leading to mutant p53 degradation. Consistent with this hypothesis, KLE cells were sensitized to BIBF1120+paclitaxel by treating with HDACi LBH589 (Figure 6(a)).

We also examined whether pathway inhibition downstream of constitutive p38 activation might circumvent the effect of the p53 GOF mutation and thereby sensitize cells to paclitaxel. We chose an inhibitor of Wee1, MK-1775. Treatment of KLE cells with Wee1 inhibitor MK-1775 in combination with paclitaxel significantly decreased cell viability as compared to paclitaxel alone, though it should be noted that we could not achieve complete cell killing with this strategy (Figure 6(b)).

## 4. Discussion

**Figure 7:**
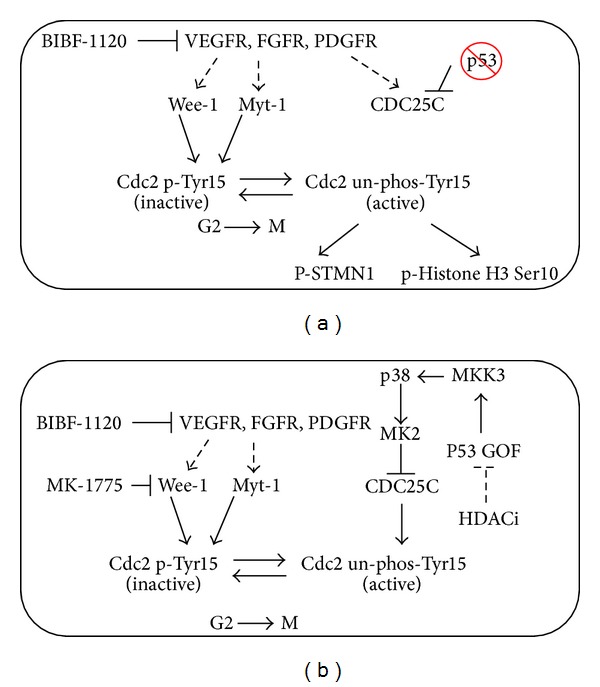
Proposed synergistic mechanisms for induction of mitotic cell death in endometrial cancer cells with mutant p53. (a) In the absence of functional p53, cells rely on the p38 pathway to maintain the G2/M checkpoint via inactivating phosphorylation of Cdc2 at Tyr15. Inhibition of RTK signaling with BIBF1120 results in premature entry into mitosis, where cells are sensitive to paclitaxel and thus undergo mitotic arrest and cell death. (b) In cells with p53 GOF mutation, the p38 pathway is hyperactivated through increased MKK3 transcription by p53 GOF. Synthetic lethality can be created by combining paclitaxel with BIBF1120 and an HDACi, which presumably disrupts the association of mutant p53 with Hsp90 and leads to its degradation. Alternatively, inhibition of Wee1, downstream of hyperactivated p38, is sufficient to restore sensitivity to paclitaxel.

While the vast majority of endometrial cancer cases will be diagnosed at an early stage, those with advanced disease remain at high risk for relapse and ultimately death from their disease. For these women a priority must be the evaluation of new agents. Our objective in this study was to identify strategies to achieve synthetic lethality based on the p53 mutational status. For endometrial cancer cells with loss of functional p53 mutation or p53-null mutation, antiangiogenesis inhibitor BIBF1120 substantially increased paclitaxel sensitivity, including in cells that have high baseline resistance to paclitaxel. This cell death was achieved through induction of mitotic catastrophe as evidenced by abrogation of the G2/M checkpoint and a high percentage of cells in M phase (Figure 7(a)). For cells with p53 GOF mutation, we identified two strategies to induce synthetic lethality to paclitaxel (Figure 7(b)). The first utilized an HDACi in combination with BIBF1120, which presumably destabilizes mutant p53. The second strategy inhibited the G2/M checkpoint controller Wee1. These data serve as a critical platform for future clinical trials in serous endometrial tumors to determine whether p53 status can be used to guide choice of therapy.

In normal and cancerous cells, WT p53 is normally expressed at very low levels. Levels rise precipitously in response to DNA damage, and WT p53 is then downregulated by MDM2. By contrast, expression of p53 GOF mutant protein is high in the absence of stress [32]. Most p53 GOF mutants fail to associate with MDM2 and instead acquire binding to new targets and protein interacting partners, such as p63 and p73 [32]. One reason for the high expression of R175H p53, as demonstrated in Figure 1, is its association with heat shock proteins, which increases its half-life [29, 30]. Another phenotype of gain of oncogenic function p53 R175H is inactivation of the Mre11/ATM-dependent DNA damage response, leading to chromosomal translocation and defects in the G2/M checkpoint [19, 33, 34]. Thus, p53 GOF mutants have acquired several key advantages that allow cells to continue to divide in the setting of stress, thereby contributing to drug resistance.

A goal of combinatorial therapy is to create synthetic lethality, where regimens are not simply additive, but synergistic. Synthetic lethality is the term for a historical genetic observation that in the presence of certain single gene mutations, blocking or mutating a second gene leads to cell death though neither mutation alone has a phenotype [35]. With respect to cancer therapy, synthetic lethality means capitalizing on the presence of a mutation in a driver protein to design novel treatments. To create therapeutic synthetic lethality, one must first know the driver mutation, understand the compensatory survival pathway which has been activated as a result of the mutation, and have an agent which can block this critical pathway. Mutations in p53, which are common in serous endometrial cancer, represent a platform upon which to design combinatorial regimens with the potential to result in tumor cell synthetic lethality. Our data provide compelling evidence that the triple angiokinase inhibitor BIBF1120 combined with paclitaxel results in synthetic lethality in p53-null tumors that are resistant to either agent alone.

The rationale for evaluating the combination of BIBF1120 plus paclitaxel for p53-null tumors is two-fold. BIBF1120 has shown promising activity in combination with chemotherapy in ovarian cancer and is currently in phase III testing in combination with a backbone of carboplatin and paclitaxel. Therefore, issues related to dosing and safety of combining this agent with chemotherapy have been addressed. More importantly, our data demonstrate that BIBF1120 exhibits significant synergy with paclitaxel in endometrial cancer cells with loss of function mutations in p53. Such cells must activate alternative pathways to maintain critical cell cycle checkpoints [15]. One of these is the p38/MK2/CDC25C/CyclinB/Cdc2 signaling cascade, which allows cells to repair DNA at G2 prior to entering M [15]. Blocking this signaling pathway completely abrogates the checkpoint in cells which lack p53 (Figure 7(a)).

To move towards better therapies, we must first achieve a new understanding of cancer biology in the hopes to identify subpopulations of patients most likely to benefit from treatment. Work from The Cancer Genome Atlas (TCGA) project has significantly improved our understanding of the genomic heterogeneity of endometrial cancers, beyond the clinicopathologic characterization commonly used of type I versus type II tumors [9]. TCGA data indicate that up to 25% of high-grade endometrioid tumors showed frequent mutations in *TP53* and extensive copy number alterations, both of which are key molecular characteristics in serous tumors. This pattern was not seen in grades 1-2 endometrioid tumors, suggesting that grade 3 endometrioid tumors were indeed more closely related to serous cancers [9]. In addition, these genomic similarities were shared between other tumors, including high-grade serous ovarian carcinoma and basal-like breast cancers insofar as these cancers share a high frequency of mutations in *TP53* (between 84 and 96 percent) and a low frequency in *PTEN*, with only 1 to 2 percent mutated. The fact that a high proportion of advanced endometrioid tumors fall into the same cluster as serous tumors suggests that these tumors should be treated similarly as serous tumors. In particular, loss of p53 in these tumors would suggest that the BIBF1120+paclitaxel regimen will induce synthetic lethality as in our studies.

In summary, our data provide clear evidence that abrogation of the G2/M checkpoint in cells with mutant p53, but not cells with normal p53, is a powerful strategy to induce synthetic lethality to paclitaxel. Future advances in the treatment of endometrial cancer must take into account genomic heterogeneity, and our data suggest a way forward by using enriched trial designs.
